# Urdu translation and validation of the International Consultation on Incontinence Questionnaire Female Sexual Matters Associated with Lower Urinary Tract Symptoms Module (ICIQ-FLUTS sex)

**DOI:** 10.12669/pjms.41.4.11425

**Published:** 2025-04

**Authors:** Anum Malik, Nuzhat Faruqui, Novera Chughtai, Urooj Kashif

**Affiliations:** 1 Anum Malik, FCPS Department of Obstetrics and Gynecology, The Aga Khan University, Karachi, 74800, Pakistan; 2 Nuzhat Faruqui, FCPS Associate Professor, Department of Surgery, Section of Urology, The Aga Khan University, Karachi, 74800, Pakistan; 3 Novera Chughtai, FCPS Assistant Professor, Department of Obstetrics and Gynecology, The Aga Khan University, Karachi, 74800, Pakistan; 4 Urooj Kashif, FCPS Assistant Professor, Department of Obstetrics and Gynecology, The Aga Khan University, Karachi, 74800, Pakistan

**Keywords:** Female sexual dysfunction, ICIQ-FLUTS sex, Urinary incontinence

## Abstract

**Objectives:**

Women all over the world suffer from urinary incontinence (UI). Female sexual dysfunction (FSD) frequently coexists with UI, which has a detrimental effect on female sexual function and frequently results in decreased desire, satisfaction, and emotional difficulties. While the ICIQ-FLUTS measures urine symptoms and the FSFI evaluates women’s sexual function, there isn’t an Urdu-validated test for UI-related sexual challenges. Our objective was to assess the validity, reliability, and internal consistency of the ICIQ-FLUTS sex for the Pakistani population, providing a clinically valuable tool for Pakistan.

**Methods:**

In a cross-sectional study conducted at Aga Khan University Hospital, Karachi, from 1st June 2024 to 15th October 2024, 58 sexually active women with UI were recruited through convenience sampling. The study involved translating the ICIQ-FLUTS sex into Urdu and evaluating its psychometric properties. Data were collected using sociodemographic and clinical questionnaires, ICIQ-UI SF, and the adapted Urdu ICIQ-FLUTS sex. Test-retest reliability was measured over two weeks.

**Results:**

Participants’ average age was 42.93 ± 8.645 years, with most being multiparous (81%) and having had spontaneous vaginal deliveries (67%). UI types included urgency (27.6%), stress (32.8%), and mixed (39.7%). The ICIQ-FLUTS sex demonstrated acceptable internal consistency (Cronbach’s alpha = 0.695), excellent test-retest reliability (ICC = 0.998) and content validity index of 1 was calculated. Concurrent validity showed a weak correlation (0.253) between ICIQ-FLUTS sex and ICIQ-UI scores.

**Conclusions:**

The Urdu version of ICIQ-FLUTS sex showed excellent reliability and content validity, satisfactory internal consistency, and acceptable psychometric properties and can be applied as a clinical assessment tool and for purposes of research to evaluate sexual problems associated with UI in Pakistan.

## INTRODUCTION

Urinary incontinence (UI) is a widespread medical issue affecting people of all ages across various countries, cultures, and ethnicities.^1^ The International Urogynecology Association (IUGA) and the International Continence Society (ICS) jointly define UI as the “involuntary loss of urine.^2^ It is a clinical condition occurring more commonly in women. Key risk factors for UI include multiple pregnancies, vaginal childbirth, menopause, low estrogen levels, diabetes, obesity, and damage to the pelvic floor muscles.^3^ Female sexual dysfunction (FSD) is characterized as a significant clinical problem that impairs a person’s ability to respond sexually or to derive sexual pleasure.^4^ It is a common health concern that encompasses issues such as decreased arousal or desire for sex, problems with orgasm, and pain in the vaginal or pelvic area during sexual activity.

Research has shown that UI often impacts women’s sexual health,^5^ leading to increased sexual abstinence, reduced desire, lower relationship satisfaction, and discomfort during intimacy.^6^ Women with UI frequently face emotional challenges, including feelings of shame, and may worry about leakage, all of which contribute to difficulties in sexual relationships.^6^ A systematic review found that UI negatively impacts sexual function.^7^ Similarly, a study in Pakistan reported a 73% prevalence of FSD among women with UI, as measured by the Female Sexual Function Index (FSFI).^8^ FSD, though acknowledged as a serious health concern, is nonetheless infrequently recognized and insufficiently treated primarily because its diagnosis and treatment rely on self-reporting. Implementing valid and reliable tools, like validated questionnaires, can help address this issue.

Several questionnaires, such as the FSFI^9^ and the International Consultation on Incontinence Questionnaire-Female LUTS (ICIQ-FLUTS),^10^ have already been translated and validated in Urdu. The FSFI evaluates various aspects of a woman’s sexual response. Meanwhile, the ICIQ-FLUTS assesses LUTS in women and their effect on Quality of life (QOL). One such tool is the International Consultation on Incontinence Questionnaire Female Sexual Matters Associated LUTS Module (ICIQ-FLUTS sex)^11^ which evaluates sexual issues related to the lower urinary tract. Its simplicity and ease of use contribute to its widespread application in clinical practice globally. ICIQ-FLUTS sex has been validated in several languages and utilized all around the world.^6^,^12^-^14^ Despite these available tools, there is currently no Urdu-validated questionnaire specifically designed to assess sexual issues related to LUTS.

Pakistan’s national language is Urdu. Translating and validating the ICIQ-FLUTS sex questionnaire for the native community was the purpose of the current study. In Pakistan, many women experience LUTS along with associated sexual issues but often hesitate to seek medical help due to discomfort and limited guidance. A self-administered questionnaire could serve as a useful tool, allowing women to report symptoms confidentially and supporting healthcare providers in evaluating and managing these conditions.

## METHODS

This research sought to evaluate the validity, test-retest reliability, and internal consistency of the ICIQ-FLUTS sex for the Pakistani population to create a valuable tool for clinical practice and scientific research in Pakistan. The Aga Khan University Hospital (AKUH) in Karachi, Pakistan’s Department of Surgery section of Urology and Department of Obstetrics and Gynecology were the sites of this study, which was conducted from 1st June 2024 to 15th October 2024. The Aga Khan University Hospital is a private hospital that provides secondary and tertiary care.

### Ethical concerns:

The institutional ethics research committee (ERC) of AKUH gave its approval to the study with the reference number 2024-9446-28323 Date: Feb. 29, 2024. Written informed consent was obtained from each participant before enrolment in the study.

### Research design, sample size, and settings:

There were two phases in the study. Phase 1 involved adapting the instrument to the Pakistani language and translating it, while Phase 2 involved assessing its psychometric qualities. Four of the eight questions of ICIQ-FLUTS sex, evaluate the presence of pain from vaginal dryness, the effect of UI on their sexual life, and pain and leakage of urine during sexual activity. The following questions assess respondents’ discomfort level on a scale from 0 (not at all) to 10 (very much). The total score ranges from 0 to 14. Higher scores indicate more severe symptoms.

### Linguistic translation and adaptation to the Urdu language:

A thorough linguistic translation that allows for the greatest degree of conceptual and technical equivalency between the original source and target languages is essential to the validation process of any measure translated into a new language.^15^ The language validation process involved the following actions.

### First Version:

The translation of the ICIQ-FLUTS sex from English to Urdu was carried out by two translators who were independent and proficient in both languages.

### Second Version:

Three specialists conducted a comparison between the Urdu and original ICIQ-FLUTS sex.

### Backward translation:

Two translators translated version two back into English. Once the experts reached a consensus, one back translation was finalized.

### Third version:

Tracking the forward and backward translation discrepancies led to its finalization.

### Final version:

The final Urdu version of the ICIQ-FLUTS Questionnaire was drafted after application on a pilot sample. A pilot study involving ten sexually active women with UI was conducted to see whether the ICIQ-FLUTS sex was understandable to the intended audience. We created the final ICIQ-FLUTS sex version in Urdu.

### Psychometric evaluation:

To test the psychometric qualities of the Urdu version of the ICIQ-FLUTS sex patients who were beyond the age of eighteen years had given their consent to participate, had a minimum one sexual encounter during the previous three weeks, and had UI were included in the study while postpartum patients, pregnant patients, who had undergone prior pelvic and anti-incontinence surgery within the six months before the study, had psychiatric diseases and cognitive impairment, illiterate in Urdu, had an active urinary tract infection, were taking medications that may affect LUTS, and had an education level below primary were excluded from the study.

The Consensus-based Standards for the Selection of Health Measurement Instruments (COSMIN)^16^ recommends a sample size based on a consensus of seven individuals per item. Since there are eight items in the ICIQ-FLUTS sex, a sample size of at least 56 was necessary. It increased to 62 when claimed inflation of 10% was taken into account for no response. Adding 10% inflation to each of the eight questionnaire items for seven patients resulted in a total of 64 individuals. Additionally, according to Sapnas and Zeller^17^ while evaluating the attributes of the health assessment tools, a minimum of 50 individuals and a maximum of 100 subjects are adequate. The urogynecology fellow obtained informed consent from all eligible patients in a private clinic room, detailing the study’s purpose, risks, and benefits in the consent form and then filled the demographics and ICIQ-UI SF questionnaire followed by filling out the Urdu version of the ICIQ-FLUTS sex questionnaire by the patient.

### Measurement instruments:

Three questionnaires were utilized to gather data: one for demographics and clinical details. The second was the ICIQ-UI SF questionnaire,^18^ which includes eight self-diagnosis elements about the circumstances of the patients’ UI in addition to four questions measuring the influence, severity, and frequency of UI. Numerical values were assigned for the analysis of their responses; the total score ranged from 0 to 21 points; the higher the sum of the points, the more severe and detrimental effects of UI on QOL. The following categories were applied to the impact on QOL: (0) no impact, (1 to 3) minor impact, (4 to 6) moderate impact, (7 to 9) severe impact, and 10 or more represents a very severe impact.^6^

The third questionnaire was the Urdu-language ICIQ-FLUTS sex questionnaire. Patients again completed the ICIQ-FLUTS sex after two weeks to assess the test-retest reliability. If no symptomatic improvements or treatments were made, patients were chosen. According to Elsman et al., the interval between repeated questionnaires should be long enough to avoid recall but short enough to ensure no clinical change, typically about one to two weeks.^19^

### Statistical analysis:

The data analysis was conducted using SPSS version 20, with descriptive statistics summarizing participant demographics and clinical features. Reliability testing included Cronbach’s alpha for internal consistency (ideally >0.80, but >0.60 acceptable)^20^ and test-retest reliability via intraclass correlation coefficient over two weeks, with values >0.70 considered good. Content validity was evaluated by an expert panel using the content validity index, while concurrent validity was assessed using Pearson’s correlation between ICIQ-FLUTS sex and ICIQ-UI SF scores to test the hypothesis linking higher scores to greater impacts on QoL and sexual function.^7^,^8^,^21^

## RESULTS

The final sample for this study consisted of 58 women after 80 were assessed for eligibility, 22 of whom were eliminated due to their lack of an active sexual life.

### Characteristics of participants:

The mean age of participants was 42.93 ± 8.65 years. Most patients were multiparous (81%), with 67.2% having delivered vaginally. Mixed urinary incontinence (MUI) was the most common type, affecting 39.7% of women (Table I). Using the ICIQ-SF questionnaire to assess the impact of UI on QoL, 17.2% of participants reported a very severe impact, and 44.8% reported a severe impact. Additionally, 48.3% experienced leakage several times daily, with 63.8% describing the amount of loss as small (Table-I).

**Table-I:** Patient characteristics.

S.No	Type of variables	Mean ±SD	Frequency (%)
1	Age	42.93 ±8.645	
2	*Parity*		
	Nullipara		4 (6.9%)
	Primipara		6 (10.3%)
	Multipara		47 (81%)
	Grand multipara		1 (1.7%)
3	Diabetes		11 (19%)
4	Hypertension		10 (17.2%)
5	*Mode of delivery*		
	SVD		39 (67.2%)
	C-section		11 (19%)
	Both		4 (6.9%)
	None		4 (6.9%)
6	History of hysterectomy		9 (15.5%)
7	*Type of incontinence*		
	Urgency		16 (27.6%)
	Stress		19 (32.8%)
	Mixed		23 (39.7%)
8	ICIQ-UI SF 3	Frequency	Percent(%)
	About once a week or less often	10	17.2%
	Two or three times a week	12	20.7%
	About once a day	8	13.8%
	Several times a day	28	48.3%
	Total	58	100.0%
9	ICIQ-UI SF 4	Frequency	Percent (%)
	Small amount	37	63.8%
	Moderate amount	18	31.0%
	Large amount	3	5.2%
	Total	58	100.0%
10	ICIQ-UI SF 5	Frequency	Percent (%)
	Mild	7	12.1%
	Moderate	15	25.9%
	Severe	26	44.8%
	Very severe	10	17.2%
	Total	58	100.0%

Among patients with MUI (16 cases), the mean ICIQ-UI SF score was 14 ± 4, and the mean ICIQ-FLUTS sex score was 3.81 ± 2.4. Patients with urge urinary incontinence (UUI) had the highest average scores on both the ICIQ-UI SF and ICIQ-FLUTS sex, indicating more severe symptoms and impact compared to those with stress urinary incontinence (SUI) or MUI. The average result for questions 2A to 5A of the ICIQ-FLUTS sex was 2.88 ±2.39, and for questions 3 to 5 of the ICIQ-UI SF, it was 12.5±3.91. The average ICIQ-UI SF score was notably higher than the ICIQ-FLUTS sex scores, indicating that the severity and impact of incontinence may not strongly affect sexual activity.

### Content validity/Face validity:

In pilot testing, ten participants completed the questionnaire, assessing its length, clarity, cultural relevance, language, and ease of understanding. All questions displayed face validity, with participants finding them clear, comprehensive, and easy to understand. Each participant completed the questionnaire independently, recognizing the importance of the questions for clinical use in Pakistan. There were no missing responses, supporting near-excellent content and face validity.

### Content validity index (CVI):

Content validity was determined by a multidisciplinary expert committee assessment. Following translation, the CVI was calculated using Waltz and Bausell’s^22^ CVI approach. Expert consensus led to the development of the questionnaire’s Urdu version. Excellent content validity was indicated by the CVI value of 1.

### Internal consistency:

Cronbach’s Alpha, a measure of internal consistency on the ICIQ-FLUTS sex questionnaire, came out to be 0.695. However, the removal of items 2A and 2B increased Cronbach’s alpha to 0.73.

### Test-retest reliability:

36 participants were used to test-retest reliability by utilizing the intraclass correlation coefficient (ICC), and the ICIQ-FLUTS sex Questionnaire showed high test and retest values (ICC = 0.998) (Table-II).

**Table-II:** Test-retest reliability.

		95% C I	
Question	ICC	Lower bound- Upper bound	Kappa
Q 2a	0.986	.973 to .993	0.944
Q 2b	0.991	.982 to .995	0.691
Q 3a	1	1.000 to 1.000	1
Q 3b	0.996	.991 to .998	0.854
Q 4a	1	1.000 to 1.000	1
Q 4b	0.999	.999 to 1.000	0.961
Q 5a	1	1.000 to 1.000	1
Q 5b	0.998	.995 to .999	0.928

Concurrent validity: By comparing the Urdu ICIQ-FLUTS sex with ICIQ-UI SF, the concurrent validity was ascertained by Pearson Correlation (0.253).

## DISCUSSION

The Urdu version of the ICIQ-FLUTS sex questionnaire demonstrated strong psychometric properties, including excellent face and content validity, robust test-retest reliability, acceptable internal consistency, and low concurrent validity. As the first validated instrument in Urdu specifically targeting female sexual concerns associated with LUTS and QoL, this tool represents a significant advancement for research in this field within Pakistan. By providing a standardized, culturally adapted instrument, this tool enables comparative studies across various Pakistani research centers, facilitating a deeper understanding of LUTS and their impact on female sexual health. The instrument is appropriate for use in regular clinical practice, epidemiological research, and treatment outcome assessment because it is straightforward, self-administered, and inexpensive. In traditionalist nations, this questionnaire can assist regulate conversations about sexual dysfunction and enhance patient care because of the social stigma associated with female sexual health.

Consistent with similar studies on the ICIQ-FLUTS sex, the mean age of participants in our study was 42.93 ± 8.65 years, aligning with the middle-aged demographic commonly studied in LUTS research. This age is comparable to that reported in the Turkish version (mean age 44.9 ± 8.9 years)^14^ and the Brazilian Portuguese version (mean age 49.1 years).^6^

The predominant mode of childbirth was vaginal delivery (67.2%), with the majority being multiparous, mirroring findings in the Brazilian Portuguese version.^6^ A study from Pakistan reported that 39% of women who delivered vaginally experienced UI,^23^ and a similar finding by another study indicated that 61.16% of women with a history of vaginal delivery also experienced UI.^24^ In our study, MUI emerged as the most common type, consistent with findings from the Brazilian Portuguese version,^6^ where 60.7% of participants had MUI. Additionally, 58.9% of participants in the Brazilian Portuguese sample reported UI several times daily, with 44.6% experiencing mild leakage, aligning with our study’s findings.^6^ The Brazilian Portuguese version reported that 82.1% of patients experienced a severe impact on QoL, corroborating a general trend in the literature that shows UI significantly impacts both specific and general aspects of QoL,^25^ as well as sexual function.^7^

The face and content validity of the ICIQ-FLUTS sex is evident across multiple language versions, with minimal missing data indicating good item comprehension. The Greek version demonstrated near-excellent validity,^12^ with no missing responses, while the Turkish and Chinese versions also showed adequate validity.^13^,^14^ These findings suggest that the instrument maintains clarity and relevance across diverse cultural settings. In terms of internal consistency, most versions of the ICIQ-FLUTS sex achieved Cronbach’s alpha values greater than 0.70, representing adequate internal consistency,^6^,^13^ with the Greek version slightly lower at 0.69.^12^ The Brazilian Portuguese, Turkish, and Chinese versions demonstrated robust internal consistency, with alpha values of 0.80, 0.863, and 0.86, respectively.^6^,^13^,^14^

The Urdu ICIQ-FLUTS sex questionnaire demonstrated excellent test-retest reliability, with an ICC of 0.998, reflecting stability in responses over time. The Brazilian Portuguese version exhibited moderate reliability (Kappa 0.36-0.76, mean of 0.59),^6^ potentially affected by participants’ interim physiotherapy between assessments. In contrast, the Greek version (Kappa 0.70-0.99),^12^ Turkish version (Kappa 0.870-0.961, ICC 0.898-0.990),^14^ and the Chinese version reported substantial to near-perfect reliability,^13^ highlighting the consistency of the ICIQ-FLUTS sex responses across different language versions. The high test-retest reliability in the Urdu and Turkish versions, in particular, suggests these versions provide highly stable data. Since the responses did not differ between the first and second stages of the questionnaire’s application, this indicates that the information gathered is consistent and that the tool can identify information and potential participant differences.^26^

The concurrent validity of the ICIQ-FLUTS sex, measured using Pearson’s correlation coefficient, showed a weak correlation with ICIQ-UI-SF total scores. This is anticipated, as the instruments assess different constructs and were not designed to measure identical outcomes. Therefore, concurrent validity for the ICIQ-FLUTS sex was primarily evaluated through hypothesis testing, underscoring the importance of the tool’s specific focus on sexual health concerns related to LUTS rather than broader urinary symptoms.

### Strengths of the Study:

Being the first study to confirm ICIQ-FLUTS sex in Urdu, it is now available to women who speak Urdu in Pakistan and other countries. The study offers a validated instrument for assessing the effect of LUTS on female sexual function in both clinical practice and research. Internationally accepted protocols were followed during the translation and validation process to guarantee conceptual and language equivalency.

### Limitations

The study’s recruitment from a single urban hospital in southern Pakistan limits its applicability to remote, rural, and diverse regions across the country. Broader validation studies are recommended to account for Pakistan’s geographic and cultural diversity. Another limitation in Pakistan was the low literacy rate, as only patients who could read and understand the questions were included. Additionally, varied interpretations of sexual intercourse due to differences in sexual orientation, beliefs, and cultural factors may have affected responses. We recommend that the questionnaire’s efficacy in tracking treatment outcomes for female sexual dysfunction and urine incontinence over time may be better understood by a longitudinal follow-up of patients

## CONCLUSION

The Urdu version of the ICIQ-FLUTS sex questionnaire demonstrates excellent reliability, strong content/face validity, and satisfactory internal consistency. This validated tool enables healthcare professionals in Pakistan to accurately assess female sexual issues related to LUTS and their impact on QOL. Moreover, it supports cross-cultural comparisons and can be used to measure treatment outcomes in both research and clinical practice. By offering a culturally appropriate and linguistically standardized tool designed for a population where female sexual health is frequently overlooked and inadequately treated, it closes a significant gap in urogynecology research.

### Authors’ Contribution:

**AM: D**esigning, collecting data, interpreting data, and drafting the article.

**NF:** Conceptualized, reviewed the manuscript, gave final approval, and is responsible for the accuracy or integrity of the work.

**NC and UK:** Did Critical review, Analysis and final approval of the manuscript.
